# Artificial Intelligence Hallucinations in Dentistry: Implications and Safeguards for Clinical Practice

**DOI:** 10.1016/j.identj.2026.109802

**Published:** 2026-08-05

**Authors:** Anand Marya, Siddharthan Selvaraj

**Affiliations:** Faculty of Dentistry, University of Puthisastra, Phnom Penh, Cambodia

## Introduction

The development of artificial intelligence (AI) has produced many new ways to operate in modern health care. These developments have also produced ways to change how we do dentistry. Recent advancements in machine learning, deep learning, computer vision, and large language model technology are contributing to the development of increasingly complex technologies that can help with diagnostic interpretation, develop treatment plans, communicate with patients, engage in educational activities, streamline administrative functions, and conduct scientific research.^1^^,^^2^ The applications of AI are now being studied across many different areas of dentistry, including oral and maxillofacial radiology, orthodontics, prosthodontics, restorative dentistry, implant dentistry, oral surgery, and oral medicine.^3^, ^4^, ^5^ Considerable potential exists for AI-based technologies in dentistry to enhance the consistency of diagnosis, improve efficiency of workflow, facilitate more individualised treatment plans, and provide more readily accessed information and support to clinicians in making clinical treatment decisions. As a result, AI now has the potential to fundamentally change how patients receive oral health care services and should not just be viewed as an adjunctive technology.

Recent progress in fields such as machine learning, deep neural networks, and large language models has had a tremendous impact on the practice of medicine by creating opportunities for activities previously deemed impossible.^2^^,^^6^ Additionally, the development of these new capabilities will allow for greater efficiency and access, increased levels of individualisation, and a greater likelihood of successful patient outcomes compared with traditional methods of providing care; however, they present a unique dilemma that many users do not clearly comprehend: the phenomenon of AI-induced hallucinations.

While interest in AI is rising, concern about AI accuracy, transparency, and trustworthiness continues to rise as well. One of the most important concerns is the existence of an “AI hallucination.” The term has proliferated throughout both science and the general public; however, its use and definition are still ambiguous or often confused with other types of errors created by AI. Strictly, an AI hallucination is defined as any output produced by AI that, although it appears to be accurate, reasonable, or credible, can be traced to information that has no basis in fact or is fabricated or unverifiable. Unlike other software errors, such as bugs, where there is usually a signal indicating a malfunction, users may not be aware of hallucinations because they are presented with language that appears confident in its presentation or produces internally consistent outputs. Therefore, inaccurate outputs may appear trustworthy, even though they do not exhibit factual truth.^7^, ^8^, ^9^

The rise in the use of generative AI in dentistry has heightened concerns over hallucination when those tools are used to make decisions affecting care, learning, or scientific communication. AI can help clinicians with diagnosis and treatment planning; students use it to learn about new procedures and techniques; researchers use it to conduct literature reviews and prepare manuscripts; and administrators can use it to document and communicate with patients. All these uses could potentially lead to errors in output that result in incorrect decisions based on those effects, sometimes without being realised immediately.^7^^,^^10^^,^^11^

AI hallucinations should be distinguished from other types of AI failure, including prediction errors, algorithmic bias, overfitting, and lack of generalisation. Although these failures are related and may all result from similar patterns, they each emerge through distinct mechanisms. For example, prediction errors occur when an AI system outputs something incorrectly despite being programmed correctly. Algorithmic bias results from training datasets that do not represent the population for which an AI program was designed to be used; thus, there are discrepancies in how well an AI program performs for different demographic and/or clinical groups. Overfitting refers to an AI program doing very well when tested using the data it trained on but doing poorly when it is tested using data it has never trained on. Lack of generalisation occurs when an AI program does not perform well when it is used outside of the environment that it was originally designed for and validated against.^12^, ^13^, ^14^

Hallucinations have 3 categories: fact-conflicting, context-conflicting, and input-conflicting.^9^ The 3 types of hallucinatory classification originated in research on natural language processing, but they can be used as a framework to understand how hallucinations may occur in dental applications.

Hallucinations may occur within a wide range of activities using generative AI in dentistry. A generative AI system may create fictitious research results, misrepresent actual research results, or recommend treatments that do not correlate with established clinical guidelines. Imaging technology-based AI may produce output that appears to be diagnostic but does not correlate with radiologic data. Educational platforms may provide inaccurate explanations of biological processes or treatment concepts, while administrative systems may generate misleading patient instructions or documentation. The diversity of these potential manifestations underscores the fact that hallucinations are not confined to a single type of AI system or clinical application. Rather, they represent a systemic vulnerability that may influence multiple components of the dental ecosystem simultaneously.^3^^,^^7^^,^^8^^,^^11^

**Fig. 1:**
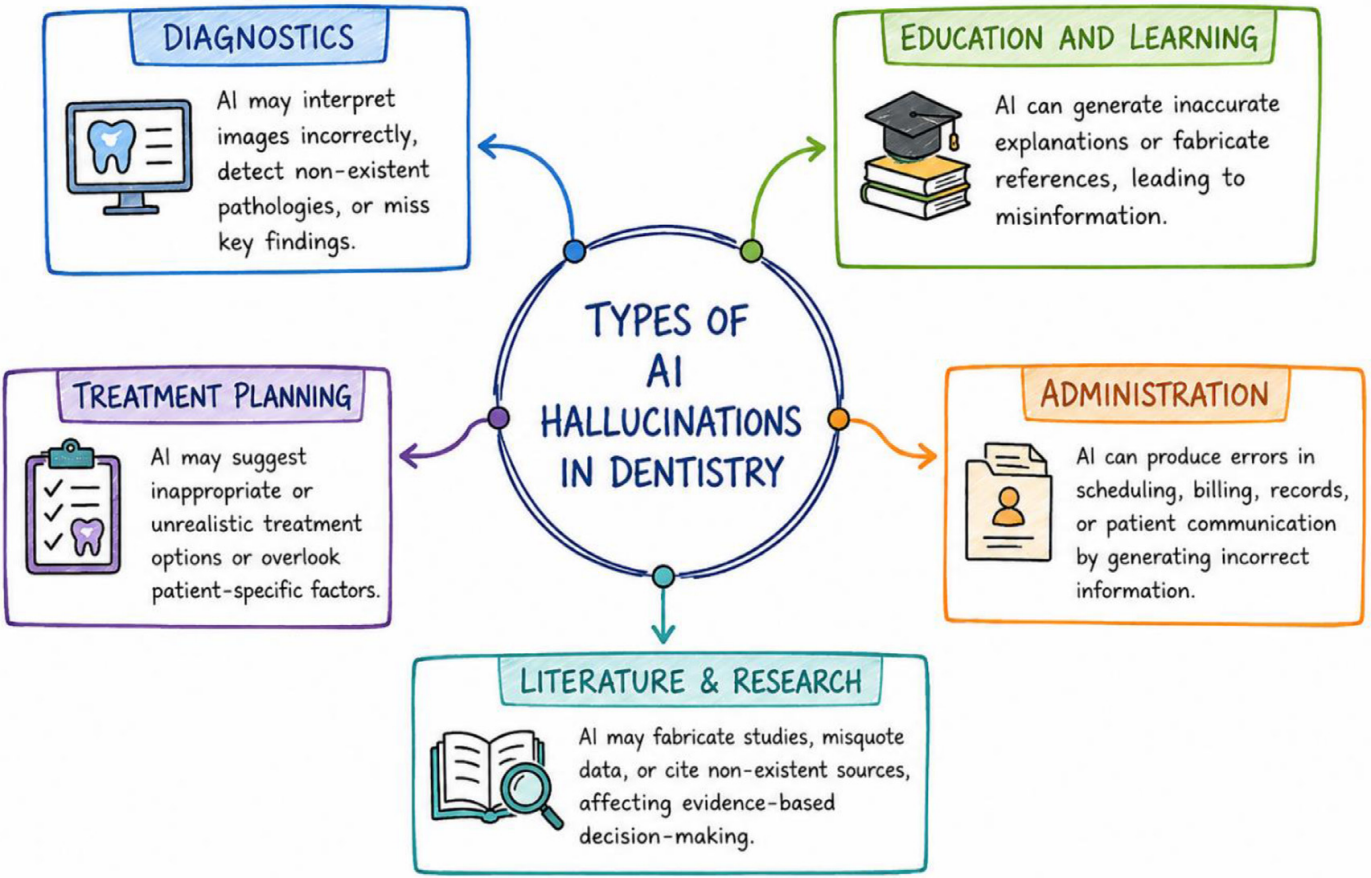
Artificial intelligence (AI) hallucinations in dentistry. The framework demonstrates how AI hallucinations may arise across multiple domains of the dental ecosystem, including diagnostics, treatment planning, research and scientific writing, education, and administrative communication.

Hallucination in AI can be described as AI outputs created by computer algorithms/procedures that are syntactically correct and contextually reasonable but incorrect, unverifiable, or made up.^2^^,^^8^^,^^9^ In contrast to “traditional” software bugs, hallucinations in AI are particularly problematic because they simulate being correct, frequently appearing to be authoritative and internally consistent with respect to language. In a field such as dentistry, where precision, evidence-based reasoning, and reproducibility are the foundation of the profession, these distortions have far-reaching implications. Figure 1 illustrates the conceptual framework of hallucinations and provides a model to categorise the domains where hallucinations occur, how they occur, the downstream effects of hallucinations, and strategies to mitigate the effects of hallucinations.

## Occurrence domains: a systemic vulnerability

AI hallucinations are not just single-event errors that occur only within a single application; they are valid examples of a systemic vulnerability affecting the entire dental ecosystem. To fully comprehend how these hallucinations can negatively affect clinical practice, the location where they occur must be taken into account.

AI systems utilise technologies in the diagnostic process, which help assess the quality of 2-dimensional radiographs, panoramic radiographs, and 3-dimensional imaging. Although it has been satisfactorily demonstrated through various studies that the sensitivity and specificity of AI deep learning systems in formal research settings are generally acceptable, their performance in everyday practice cannot be considered equivalent. The reasons for this include (1) a mismatch between anatomic structures and anatomic landmarks and (2) the presence of imaging artefacts. Errors in identifying anatomic landmarks may lead to misdiagnosed caries or artefacts that are not indicative of disease. Since these types of errors are likely to be made by AI systems based solely on imaging data, clinicians may inadvertently rely on the output of AI as being accurate without understanding the level of uncertainty associated with the output. Therefore, clinicians could be led to make poor decisions regarding the accuracy and validity of a diagnosis, which could ultimately influence clinical judgement.^5^^,^^14^, ^15^, ^16^, ^17^

Treatment planning presents an even more difficult challenge than diagnosis. AI is moving towards the ability to create orthodontic setups, sequencing of restorative treatment, and strategies for implant placement. In this case, hallucinations may include biologically implausible tooth movements, inappropriate appliance selection, or unrealistically short treatment timelines. However, unlike diagnostic errors that can be resolved by obtaining additional images or performing additional tests, errors in the treatment planning process have a cascading impact on all aspects of patient care. This is especially true in orthodontics, where biomechanical accuracy and long-term treatment stability are critically important.^18^, ^19^, ^20^, ^21^

The number of academic studies relying on generative AI technology to facilitate the writing process and create literature reviews has rapidly increased. These tools establish efficiencies in scientific writing but introduce risks such as fabricated citations, misinterpretation of study findings, and creation of false scientific narratives.^7^^,^^8^^,^^10^^,^^11^ Fabrications of this nature may undermine the credibility of scientific communication and create misleading narratives that become disseminated throughout the academic community. Furthermore, the increasing number of AI-assisted manuscripts contributes to additional challenges during peer review and increases the likelihood that fabricated or inaccurate information remains undetected.

## Clinical implications of AI hallucinations in dentistry

With the rapid adoption of AI into clinical practices, awareness of the potential consequences of AI-generated hallucinations within patient care is critical. Unlike traditional software failures, which may produce clear indicators of failure, hallucinated outputs are frequently logical, scientifically sound, and clinically reasonable. As such, they raise significant concerns for health care practitioners due to their potential to be accepted by the health care practitioner as accurate and reliable, particularly when AI-generated suggestions are consistent with health care practitioners’ existing beliefs and preconceptions. As AI technologies show great potential for improving patient diagnostic accuracy, treatment efficiency, and clinical decision-making, hallucinated outputs represent a new category of risk that may affect clinical care decisions at various points throughout the patient care continuum.^22^, ^23^, ^24^

Depending on the type of AI involved, the implications of hallucinations in the clinic are different. Generative AI systems can create false treatment recommendations and false references to science, misrepresent guidelines in clinical practice, or generate uninformed conclusions regarding how to manage patients. In contrast, computer vision systems may misclassify images taken by radiography or misinterpret other images in a clinical setting. While the mechanisms for AI error vary, both types of AI mistakes have the potential to impact diagnosis, treatment planning, and patient outcomes. The real issue is not just that AI generates mistakes on occasion but how frequently an AI’s prediction of its output’s accuracy is of such high confidence that users would not verify it independently.^7^, ^8^, ^9^

Diagnostic applications represent one of the most extensively studied areas of AI implementation in dentistry. Deep learning systems have demonstrated encouraging performance in detecting dental caries, periodontal bone loss, periapical pathology, impacted teeth, and various craniofacial abnormalities.^15^, ^16^, ^17^^,^^25^ Several systematic reviews have reported diagnostic performances approaching those of experienced clinicians under controlled research conditions.^3^, ^4^, ^5^ Nevertheless, these systems remain vulnerable to inaccuracies when confronted with situations that differ substantially from their training datasets. Variations in image quality, anatomic complexity, imaging artefacts, and population-based characteristics may all result in incorrect output by AI systems. Metallic restorations and artefacts due to beam hardening, together with unusual anatomic structures, may lead to misinterpretation of normal structures as being pathologic or missing genuine pathology on occasion. Such inaccuracies may appear minor in isolation but will affect confidence in diagnosis and subsequent clinical decisions should they be accepted without independent verification.^5^^,^^14^

The consequences of hallucinations become even more significant when they occur during treatment planning. While diagnostic inaccuracies can often be fixed by follow-up physical assessments or tests, errors made in the treatment planning phase could impact many later clinical decisions over time. AI-based planning tools are being increasingly integrated into orthodontic, prosthodontic, implant, and restorative disciplines; these tools can provide suggestions for sequencing treatments, device types, implant locations, type of material used for devices, and predicted treatment results. Evidence could be generated by an AI system that suggests impossible biological tooth movements for orthodontic purposes, unjustified extraction recommendations, predicted treatment times that are unreasonable, and so on. If these recommendations are accepted without question, they could result in improper treatment strategies and harm to patients.^18^, ^19^, ^20^, ^21^^,^^25^^,^^26^

The impact of hallucinations in prosthodontics and implant dentistry is particularly significant because treatment requires many specialties to work together. For example, an AI implant planning system might create false estimates of bone density and/or predict the time it will take for a dental implant to join with the bone, based on no scientific evidence to support the AI because the AI was developed without any kind of proof that this was possible; these false estimates could support an early loading protocol or create false expectations on the final outcome of the treatment. Similarly, a generative AI rehabilitator for a complete mouth restoration may propose occlusal concepts, articulating device settings, or restoration concepts that are not only unsupported by scientific evidence but also inappropriate for an individual patient. Within digital dentistry workflows, hallucinated information regarding milling tolerances, cement space parameters, or material properties may influence computer-assisted design/computer-assisted manufacturing design processes and ultimately compromise restoration fit, function, or longevity. AI systems that generate fabricated performance characteristics for restorative materials may likewise influence material selection decisions and expose patients to an increased risk of restoration failure.^18^, ^19^, ^20^, ^21^^,^^25^^,^^26^

The use of hallucinations in orthodontics has similar clinical significance to those seen above. Many new AI systems can now generate predictions about how and when to perform orthodontic therapy, predict how well the therapy will work, and help clinicians in developing a treatment plan. However, these same AI-generated predictions may have hallucinated outputs, such as unrealistic tooth movement speed, unrealistic anchorage predictions, or treatment plans that do not consider biological limitations or biomechanics; if the experienced clinician does not identify and correct the inaccuracies created by the AI, the same AI-generated predictions would result in a longer period of time to complete treatment, less efficient treatment, and/or an increased risk of root resorption and/or instability following treatment completion.^21^

In addition to direct clinical uses, AI hallucination may also affect scientific inquiry and academic communication. The proliferation of generative AI tools in literature reviews, manuscript writing, grants, and other forms of scientific communications has created a number of efficiencies while jeopardising the integrity of scientific works. There have been countless examples of instances where an AI system created fabricated references, invented DOIs, misrepresented research findings, or created misleading summaries of evidence.^7^^,^^8^^,^^10^^,^^11^ Because these outputs are generated in an extremely confident and authoritative manner, they will frequently go unnoticed during the manuscript and peer-review process. These types of inaccuracies may also detract from the validity of evidence-based practice and help to propagate misinformation throughout the scientific community. As a result, any AI-generated material used in a scientific paper should be thoroughly verified against the original source material before being made available to the public.^7^^,^^8^^,^^10^^,^^11^

Hallucinations can affect the way students look at an educational environment—specifically, when dental students are given ways to seek support in their educational environment, such as through AI-powered tutoring, educational software, and virtual learning tools. If used properly, these resources are beneficial and allow students to more easily access information, improve the way they can learn on their own, and ultimately make the process of receiving an education more efficient. Unfortunately, it is also very likely that students will come across hallucinated explanations, made-up scientific facts, or false recommendations for clinical procedures from the use of AI tools. As a result, when students use the information they gather from these tools to build a knowledge base, they may inadvertently develop it with false information. One major area that could create a problem in future clinical education and practice is that by overrelying on AI-generated outputs, students may develop a limited ability to conduct independent critical appraisal and/or an independent evaluation of evidence. As students continue to increasingly rely on AI-generated outputs for the development of clinical reasoning, they may lose the necessary skills for safe and effective clinical treatment in the field of dentistry. Recent activities related to the creation of AI competency frameworks for use within dental education have highlighted the need to ensure that all future dental graduates not only learn how to effectively utilise AI for learning but also have an understanding of how AI limitations exist and can affect clinical judgement, how to identify hallucinated outputs, and how to perform critical evaluation of AI-generated outputs when making decisions.^1^^,^^27^^,^^28^

AI hallucinations have broader consequences than individual mistakes; they can lead to behavioural and cognitive changes that are significant. Repeated exposure to faulty AI output can cause clinicians to develop different ways of interpreting information and making clinical decisions. In addition to increasing clinicians’ acceptance of incorrect AI-generated recommendations through automation bias, confirmation bias can mean that someone will tend to accept AI output that supports a preexisting belief while ignoring evidence that contradicts that belief. All of these cognitive issues will increase how much AI hallucinations influence individual clinicians and increase the risk of poor information being used to make decisions about clinical care. Therefore, the challenge of AI hallucinations needs to be regarded as more than a technology issue and will require an educational, clinical, ethical, and regulatory response. Human oversight, critical thinking, and professional judgement should remain the primary focus in all applications of AI in dentistry so that technological innovation only enhances patient care, rather than detracts from it.^12^^,^^13^

Broader implications of AI hallucinations go beyond inaccurate single errors and include behavioural, cognitive, and decision-making factors. If clinicians regularly see inaccurate information from AI, it will in time alter the way that clinicians interpret data and make decisions. The phenomenon of automation bias, or placing too much trust in an automated system, would encourage a clinician to accept AI recommendations without fully vetting them prior to use. Additionally, confirmation bias will lead an individual to accept AI outputs that support their prior beliefs while ignoring those that contradict their belief system. Such cognitive phenomena would compound the effects of hallucinations and further increase the likelihood that the use of inaccurate AI information will impact patient care outcomes.

Therefore, AI hallucinations must be seen not only as a technological concern but also as a multidimensional problem that will require an educational, clinical, ethical, and regulatory response. The utmost importance must be placed on critical thinking, human oversight, and professional judgement, ensuring that all AI applications in dentistry enhance patient care rather than reduce its quality.^12^^,^^13^

## Education and learning environments: the impact of hallucinatory AI

The influence of hallucinations in educational and learning settings is pervasive. Within dental curricula, for example, AI-based tutoring platforms, automated assessments, and/or virtual simulations are part of everyday operations. If students use an AI-based tutoring platform or an automated assessment tool and receive a “hallucinated” explanation or a “hallucinated” correct response, it can become part of the student’s daily learning experience, leading to fewer opportunities for the student to develop the foundational knowledge or critical thinking skills required to validate their use of clinical reasoning and/or to validate their ability to use knowledge independently.^27^^,^^29^^,^^30^

As AI tools become more prevalent in administrative and communication settings, the use of AI tools for patient communication is increasing. Thus, any errors caused by AI tools in an administrative or communication environment have the potential to become legally, financially, and ethically problematic as patient safety and trust may be eroded due to cumulative errors. The cumulative impact of all these errors in the administrative and communication settings will affect the way health care is delivered to all patients and trust in the health care system as a whole.^22^^,^^24^

## AI hallucinations in low- and middle-income countries: an emerging equity challenge

Concerns regarding “AI hallucinations” apply across all health care systems, but their potential to impact low- and middle-income countries (LMICs) may be particularly pronounced. All of the current AI systems used in health care and dentistry have been developed and trained using datasets predominantly from high-income countries, where incidences of disease, health care setup and delivery, treatment philosophies, demographics, and access to health care differ substantially from those in resource-constrained countries. Therefore, models that perform well in their development and validation may not have the same accuracy and reliability when being applied to populations that were underrepresented during training.^14^^˒^^22^^˒^^24^

Dentistry is particularly challenged by the lack of standardisation in various aspects, such as differences in the overall amounts of oral disease, differences in the socioeconomic aspects of health and oral health care, differences in cultural practices and beliefs regarding oral care, differences in diets, and differences in ways to provide health care. AI models created using datasets primarily from North America, Europe, and/or East Asia are not likely to resemble what and how we find care to be in regions that are classified as LMICs, such as Southeast Asia, Africa, and Latin America. As a result, AI applications operating with unvalidated datasets within LMIC settings are likely to produce inaccurate results at an alarming rate. As a result, these inaccuracies have the potential to further the inequities that currently exist in oral health care systems by providing less than reliable decision support to underserved populations who already experience limited access to specialist care and advanced diagnostic services.^21^, ^22^, ^23^

Along with problems having to do with their clinical utilisation, developing countries have begun implementing generative AI tools into teaching and learning in many institutions of higher education. However, classes on AI literacy are usually not readily available to students/teachers, thus making it increasingly difficult for them to understand hallucinated content and critically evaluate any information produced by generative AIs. This results in misinformation produced by generative AIs being considered valid and accurate by students/teachers, leading to the possibility of creating adverse educational outcomes for both parties. Similar challenges exist for researchers attempting to verify their findings because there are usually no available resources to independently verify the literature summaries, references, or scientific interpretations of the scholastic outputs produced by generative AIs.^23^^,^^29^

These issues will be remedied through significant efforts to create representative and inclusive dental AI. Some of the things that should be included in future projects are the creation of region-specific datasets, multiple research sites collaborating on a single project, and the creation of multicountry projects to validate findings across a variety of nations. Additional investment into open-source models will help provide access to the latest high-end technology for underserved areas, as well as encourage transparency and reproducibility. Open-source models also allow for adaptation and validation to local needs, enabling LMIC researchers and dentists to test AI tools before deploying them broadly in their clinical practice. Developing a framework for unbiased AI utilisation will require all countries’ commitment to equality, equity, and ethical application of innovative technologies.^21^^,^^30^

## Balancing risks and benefits: why AI still matters

Many substantial benefits have already been shown through the application of AI technologies within numerous areas of dentistry, and it is essential to provide a balanced viewpoint so as not to create the perception that hallucinations warrant a rejection of AI altogether. Rather, hallucinations should be viewed as a manageable shortcoming to an otherwise revolutionary technology.^3^^,^^4^^,^^17^^,^^31^^,^^32^

The past decade has seen incredible advancements in AI systems’ capability to accurately detect dental caries, measure periodontal bone loss, diagnose periapical pathology, identify oral lesions, and diagnose craniofacial abnormalities.^3^, ^4^, ^5^^,^^15^, ^16^, ^17^ The diagnostic performance of AI systems has also been reported to be comparable with that of experienced clinicians, particularly when AI is being used in conjunction with human clinical judgement.^3^, ^4^, ^5^ AI-assisted workflows have also improved efficiency in the areas of radiographic interpretation, treatment planning, scheduling appointments, completing administrative tasks, and communicating with patients.^17^^,^^19^^,^^25^ Collectively, these advancements will reduce clinician workloads, increase clinician productivity, and increase patients’ access to care.

AI-based tutoring systems, adaptive learning platforms, and virtual simulation technologies provide opportunities for personalised learning and competence development in the educational setting. Recent discussions in the dental education community highlight the need for dental professionals of the future to be prepared to work collaboratively with AI technology in the same way that past generations have done so with digital radiography, computer-assisted design/computer-assisted manufacturing technologies, and 3-dimensional imaging.^1^^,^^28^^,^^33^

Consequently, it is the intention of AI to advance the education of dental professionals by providing them with knowledge of the advantages and disadvantages of using AI. Similarly, generative AI has previously been useful for producing scientific papers, editing language, and extracting information through retrieving knowledge and synthesising information. When proper supervision and validation are performed, these tools can improve academic/research productivity and improve access to knowledge. The issue with generative AI is not the generative AI technology but how to ensure that what has been generated by a generative AI is supported and validated by verifiable evidence before incorporating it into clinical practice, educational materials, or scientific publications.^7^^,^^8^^,^^10^^,^^11^^,^^34^

Additionally, advancing the future of AI will depend on scientific and technological advancements, along with a well-defined governance framework and processes that both limit the potential downsides and enhance the potential benefits associated with AI and its application within the field of dentistry.

## Towards safer AI: mitigation and governance strategies

Collaborating across all types of technology innovations, educational programs, clinical practices, and legal regulations is essential to achieve a common target: minimising the impact of AI hallucinations. To ensure that AI does not produce hallucinations, there will be many different ways in which to accomplish success and guarantee that they never take place. Hence, many different entities will have to pursue numerous independent protections or safeguards through a total life cycle of AI in an integrated manner.

One of the first and most critical strategies that should be employed in mitigating AI-generated hallucinations is developing AI literacy in dental health care practitioners. Due to the increasing presence of AI technologies in routine practice, dental clinicians will need a thorough understanding of how those technologies work, their limitations, and when hallucinations are most likely to occur within the context of practice. As such, training sessions on AI should teach dental clinicians about essential machine learning concepts as well as how to critically evaluate AI-generated outputs. As evidence of AI becoming a core competency in dentistry, many organisations have started advocating for competency-based education on AI in dental schools.^1^^,^^28^

Rigorous validation prior to clinical deployment of AI solutions is a second priority. Validation should be conducted in a manner that accounts for variability among patient groups, practice settings, and geographical areas, in addition to being conducted in controlled research settings. Additionally, continued monitoring of performance following deployment is critical since clinical practice, patient demographics, and the health care system’s evolution can impact AI performance. External validation studies and precise description of research findings will assist the medical community in recognising the capabilities and limitations of AI systems entering clinical use.^14^^,^^33^^,^^35^

The third pillar of safe AI use is focused on transparency and explainability of AI resources. Clinicians are able to identify inaccurate outputs better when they understand how a recommendation was made. As such, explainable AI methods seek to provide insight into the underlying reasoning processes that led to the AI producing its recommendations, ultimately leading to greater reliance on AI, improving clinician accountability, and providing clarity to clinicians as to how to understand when to use the AI-generated recommendation. Although more research is needed to establish the best methods for increasing explainability, increasing transparency may continue to help reduce excessive trust in the use of automated recommendations and further encourage critical analysis of AI information.^12^^,^^13^^,^^36^

Maintaining oversight by humans is also essential. Rather than replacing clinicians’ expertise, AI is intended to support the judgement of the clinician to make diagnoses, develop treatment plans, communicate with patients, and make professional decisions. Human-in-the-loop models involve the clinician reviewing and validating AI-generated content prior to implementing it, thereby adding a layer of protection against the potential of AI hallucinations and other errors by AI.^6^^,^^12^

We must also develop ethical and regulatory frameworks to keep pace with technological advancements in AI. Guidance on the responsible use of AI in health care is being developed by international standards development organisations, professional organisations, regulatory bodies, and academic institutions. Recommendations include but are not limited to transparency, accountability, protection of patient privacy, algorithmic fairness, and the disclosure of the use of AI in both scientific writing and clinical decision-making.^13^^,^^35^^,^^36^ Additionally, as AI continues to develop, regulatory oversight will become more important to ensure that innovations proceed in ways that preserve patient safety and the trust of the general public.

## Reducing negative impact on patient care

In practice, hallucinated outputs will cause clinicians to make incorrect clinical decisions related to diagnostic error, treatment selection error, and/or failure to recognise patient complications. The possibility of an error occurring is increased by cognitive biases, such as automation bias or confirmation bias.

The 5 main parts of how to provide solutions for reducing the chance that these tools will have a negative impact on patient care are listed as follows:

1. Train dentists on the use of AI tools: AI tools should be included in graduates’ training as part of dentist education. This will educate dentists about what AI can and cannot do, how to recognise errors, and how to use critical thinking to analyse and utilise AI data.^1^^,^^27^^,^^28^
2. Validation of AI tools: Dental professionals need to validate that AI tools can be trusted, and developing AI tool validation standards is needed. For AI tools to be relied upon, those tools must be clinically validated through benchmarks and ongoing performance monitoring in diverse populations.^14^^,^^33^^,^^35^
3. Deliver transparency and explainability: An AI tool must be transparent. This will allow dentists to see what they have provided for processing by AI and why it has been processed in a particular manner to identify any mistakes that the AI system may have made.^36^, ^37^
4. Human decision-making must remain at the center of AI use: Early on, AI should be utilised to support clinical decision-making but not as a replacement for clinical judgement.^6^^,^^12^^,^^13^
5. Develop ethical and regulatory policies and controls: To promote responsible use of AI tools in dental practice, institutions must develop ethical guidelines for handling AI tools in practice, set regulations regarding AI tools in clinical situations, and put safeguards/controls in place for the development/editing of evidence-based literature that utilises AI tools.^1^^,^^13^^,^^31^^,^^32^^,^^35^

## Conclusion

The future of dentistry is being dramatically impacted by the rise of AI technology, with limitless potential to increase diagnostic accuracy, improve clinical workflow efficiency, provide optimal personalised treatment plans, enhance methods for delivering education, and facilitate rapid advances in scientific knowledge. However, one important issue that has been all but ignored in the dental literature is the phenomenon of AI hallucinations. Due to their nature, AI hallucinations have the potential to affect decision-making related to clinical care, education, scientific publishing, and policy development in ways that will not be apparent to many users.

This article proposes that AI hallucinations should not be viewed simply as isolated technological deficiencies but rather as manifestations of larger issues related to data quality, the design of the AI model, algorithmic bias, interpretability, human impact, and governance. The implications of this issue for the different areas of dentistry cannot be overstated. AI hallucinations may be even more consequential for developing economies, where there is lower representation in AI training data and less access to validation resources, leading to higher susceptibility to AI-generated data.

Recognising the risk associated with hallucinations does not detract from the major benefits that AI currently provides. In fact, it highlights the need for implementing responsible strategies to maximise the potential benefits and minimise any potential adverse effects on practice. These strategies must include validation, transparency, education, and human oversight. The future of AI in dentistry will be built through collaboration between clinicians, educators, researchers, developers, regulatory bodies, and professional organisations. Leveraging cohesive governance structures, creating competency-based education curricula, designing explainable systems, and developing equitable validation approaches will allow the profession to derive maximum value from AI while minimising potential harms.

As dentistry continues to enter an AI-enabled future, critical thinking, evaluation of evidence, and professional judgement must be maintained as core competencies at the point of clinical care. AI is not intended to replace humans; rather, it is meant to augment existing knowledge/skills and provide dentally based practitioners with an additional source of information. When implemented properly, AI can be one of the most valuable and impactful resources in contemporary dental practice; however, whenever using AI, all derived outputs should be interpreted within the framework of sound clinical judgement and factual data.

## Author contributions

Anand Marya: Conceptualisation; Methodology; Investigation, Writing − review & editing; Siddharthan Selvaraj: Writing − original draft and visualisation. All authors have read and approved the final version of the manuscript.

## Conflict of interest

None disclosed.
